# Gründe für die Ablehnung behördlicher Empfehlungen und Maßnahmen zum Schutz vor SARS-CoV-2 – eine qualitative Studie auf Basis von Beiträgen in sozialen Medien

**DOI:** 10.1007/s00103-021-03315-y

**Published:** 2021-04-14

**Authors:** Diana Wahidie, Yüce Yılmaz-Aslan, Sabahat Ölcer, Tuğba Aksakal, Patrick Brzoska

**Affiliations:** 1 Lehrstuhl für Versorgungsforschung, Fakultät für Gesundheit/Department für Humanmedizin, Universität Witten/Herdecke, Alfred-Herrhausen-Straße 50, 58448 Witten, Deutschland; 2 Fakultät für Gesundheitswissenschaften, AG3 Epidemiologie & International Public Health, Universität Bielefeld, Bielefeld, Deutschland; 3 Fakultät für Gesundheitswissenschaften, AG6 Versorgungsforschung und Pflegewissenschaft, Universität Bielefeld, Bielefeld, Deutschland

**Keywords:** Corona, Dokumentenanalyse, Qualitative Studie, COVID-19, Schutzmaßnahmen, Soziale Medien, Corona, Document analysis, Qualitative study, COVID-19, Protection measures, Social media

## Abstract

**Hintergrund:**

Um die Ausbreitung von SARS-CoV‑2 (schweres akutes Atemwegssyndrom-Coronavirus-Typ 2) zu verlangsamen, haben Bund und Bundesländer Schutzmaßnahmen ergriffen, die weitreichende Folgen für die Bevölkerung haben. Diese Maßnahmen umfassen u. a. die zeitweise Einschränkung des Betriebs von Freizeiteinrichtungen sowie Kontakt- und Reiseeinschränkungen. Die Maßnahmen rufen gemischte Reaktionen hervor, wobei Teile der Bevölkerung Empfehlungen und Vorgaben ignorieren.

**Ziel der Arbeit:**

Ziel der vorliegenden Studie ist es, auf Basis der Beiträge in sozialen Medien die Gründe für die Ablehnung von Schutzmaßnahmen zu untersuchen.

**Material und Methoden:**

3 soziale Netzwerke (Facebook, Twitter und Youtube-Kommentare) wurden für den Zeitraum 02.03. bis 18.04.2020 systematisch hinsichtlich der Einstellungen zu Kontaktbeschränkungen und anderen Schutzmaßnahmen mittels qualitativer Dokumenten- und Inhaltsanalyse untersucht. Insgesamt wurden 119 Beiträge in die Analyse aufgenommen und interpretiert.

**Ergebnisse:**

6 Hauptkategorien und 4 Unterkategorien wurden im Zusammenhang mit der Ablehnung der Schutzmaßnahmen identifiziert: Fehlinformationen der sozialen Medien (Verharmlosung und Zweifel an der Wirksamkeit), Misstrauen gegenüber den etablierten öffentlichen Medien, Wissensdefizite und Verunsicherung, Einschränkung der Grundrechte, die Rolle der Behörden (Bevölkerungskontrolle und mangelndes Vertrauen in das Robert Koch-Institut) sowie wirtschaftliche Auswirkungen der Pandemie.

**Diskussion:**

Fehlinformationen in sozialen Medien und Wissensdefizite können zu einer Unterschätzung der Pandemie beitragen. Zudem können wirtschaftliche Belastungen mit der Ablehnung von Schutzvorkehrungen einhergehen. Zur Erhöhung der Akzeptanz implementierter Schutzmaßnahmen stellen Gesundheitsaufklärung sowie transparente und evidenzbasierte Kommunikation relevante Determinanten dar.

## Abstract (en)

**Background:**

In order to slow down the spread of SARS-CoV‑2 (severe acute respiratory syndrome coronavirus type 2) the federal states and the government in Germany have implemented protective measures with far-reaching consequences for the population and the economy. Amongst others, these measures include the temporary restriction of the operation of leisure facilities as well as contact and travel restrictions. These government regulations and recommendations have provoked mixed reactions, with some parts of the population not complying accordingly.

**Objectives:**

The aim of the present study is to explore reasons for the noncompliance with protective measures on the basis of social media posts.

**Materials and methods:**

Three social networks (Facebook, Twitter, and YouTube comments) were systematically investigated for the period 2 March to 18 April 2020 with regard to attitudes and beliefs about “social distancing” and other protective measures by means of qualitative document and content analysis. A total of 119 postings were included in the analysis and interpreted.

**Results:**

Six main categories and four subcategories were identified in terms of the rejection of protective measures: misinformation of social media (trivialization and doubts about the effectiveness of protective measures), mistrust of the established public media, knowledge deficits and uncertainty, restriction of basic rights, the role of authorities (population control and poor trust in the Robert Koch Institute), and economic consequences of the pandemic.

**Conclusion:**

Misinformation in social media and knowledge deficits may contribute to underestimating the pandemic. In addition, economic pressures may contribute to rejecting protective measures. To increase the acceptance of implemented protective measures, health education and transparent and evidence-based communication represent relevant determinants.

## Einleitung

Die COVID-19-Pandemie (Corona Virus Disease 2019) stellt eine der größten gesellschaftlichen Herausforderungen seit Ende des Zweiten Weltkriegs dar und bringt für viele Menschen zahlreiche Veränderungen mit sich. Bis zum 14.03.2021 waren weltweit über 119 Mio. bestätigte Infektionsfälle und über 2,6 Mio. COVID-19-bedingte Todesfälle zu verzeichnen [1]. In Deutschland wurde der erste Infektionsfall mit SARS-CoV‑2 (schweres akutes Atemwegssyndrom-Coronavirus-Typ 2) am 28.01.2020 bestätigt [2]. Seitdem hat sich SARS-CoV‑2 in allen Bundesländern verbreitet [3]. Bis zum 14.03.2021 haben sich in Deutschland über 2,5 Mio. Menschen mit SARS-CoV‑2 infiziert und 73.371 Menschen sind bisher an COVID-19 verstorben [4].

Zur Eindämmung der COVID-19-Pandemie in Deutschland haben Bund und Länder Mitte März 2020 unterschiedliche Maßnahmen zur Beschränkung des öffentlichen Lebens ergriffen [5], die bis dahin unbekannte Einschnitte in die Freiheit vieler Menschen darstellten. Diese Maßnahmen umfassten beispielsweise Kontaktbeschränkungen (Social Distancing), Reisebeschränkungen, die Schließung von Bildungseinrichtungen sowie die Einschränkung des Betriebs von Hotels, Gaststätten und Freizeiteinrichtungen [6, 7]. Maßnahmen wie diese verfolgen das Ziel, die SARS-CoV-2-Infektionsrate zu reduzieren und damit die Überlastung des Gesundheitssystems zu verhindern [8].

In der Bevölkerung rufen die genannten Schutzmaßnahmen und Empfehlungen gemischte Reaktionen hervor. Während zwar große Bevölkerungsteile die Maßnahmen befürworteten [9], kamen auch zahlreiche Personen den Empfehlungen und Verordnungen nicht nach und hielten z. B. empfohlene Abstände und Kontaktbeschränkungen nicht ein [10].

Mit zunehmender Zeitdauer, in der die Schutzmaßnahmen Bestand hatten, wurde die Ablehnung teilweise noch größer und spiegelte sich beispielsweise in, teils nicht genehmigten, Demonstrationen unter Missachtung geltender Abstandsregeln wider [11]. Dies lässt sich auch anhand von Ergebnissen der Befragungswellen 5 bis 10 der COSMO-Studie (COVID-19 Snapshot Monitoring) bestätigen, in denen ein Großteil der Befragten zwar den restriktiven Maßnahmen zustimmt und bereit ist, diese zu befolgen, jedoch im Vergleich zu den vorherigen COSMO-Wellen ein kontinuierlich sinkender Trend in der Akzeptanz der Schutzmaßnahmen zu verzeichnen ist. Dieser „Ermüdungseffekt“ im Zusammenhang mit der Akzeptanz der Maßnahmen geht mit einer geringeren Sorge vor der Überlastung des Gesundheitssystems sowie einem steigenden Bedürfnis nach der Veranstaltung von Demonstrationen einher [12, 13].

Das psychologische Konzept der Reaktanz kann als Erklärung für die Ablehnung behördlicher Maßnahmen herangezogen werden. Mit „Reaktanz“ wird die Motivation zur Wiederherstellung von Freiheitsspielräumen bezeichnet. Wenn die Maßnahmen als Einschränkung der persönlichen Freiheiten empfunden werden, kann dies die Ablehnung gesundheitsbezogener Empfehlungen zur Folge haben. Dadurch kann es zu unerwünschten Effekten, beispielsweise einem gefährdenden Risikoverhalten, kommen, indem die Gesundheitsbotschaft das Gegenteil des ursprünglich intendierten Verhaltens bei den Empfängern hervorruft [14]. Gesundheitsappelle können zudem als Bedrohung wahrgenommen werden, da sie durch die Konfrontation mit der eigenen Verletzlichkeit negative Gefühle hervorrufen können [15].

Subjektiv bedrohlich empfundene Botschaften können wiederum Abwehrreaktionen in Form von Verdrängung und Verharmlosung risikobehafteten Verhaltens hervorrufen [16]. Dies kann durch die Theorie der kognitiven Dissonanz erklärt werden, der zufolge Menschen ein Gleichgewicht in ihren Wahrnehmungen anstreben („Konsonanz“) und Zustände vermeiden, die diesen entgegenstehen („Dissonanz“; [17, 18]).

Soziale Medien sind bei der öffentlichen Reaktion auf eine Pandemie und bei der Verbreitung von Informationen in pandemischen Krisensituationen von großer Bedeutung. Sie haben sich als zentral für die Meinungsbildung im Kontext der Pandemie und der damit verbundenen Auswirkungen erwiesen. Insbesondere aufgrund der Maßnahmen zur sozialen Distanzierung ist das Vertrauen in die Kommunikation durch soziale Medien gestiegen [19, 20]. Die globale Ausbreitung von SARS-CoV‑2 hat jedoch auch eine Flut an Informationen („infodemic“) zur Folge, die teils richtig und teils falsch sind [21]. Die wachsende Zahl von Verschwörungstheorien und Falschinformationen kann die Risikowahrnehmung der Bevölkerung senken und somit zu einer Ablehnung behördlich angeordneter Maßnahmen beitragen [22–24].

Anders als zu Beginn der Pandemie erhofft [2], ist, Stand März 2021, davon auszugehen, dass SARS-CoV‑2 viele Staaten der Welt trotz positiver Entwicklungen im Bereich der Verfügbarkeit von Impfstoffen noch über einen langen Zeitraum beschäftigen wird. Dabei ist mit einem wellenförmigen Infektionsverlauf zu rechnen [25]. Dieser wird es erlauben, in Phasen mit geringer Infektionsrate Schutzmaßnahmen zu lockern, aber auch erfordern, bei einem Anstieg der Infektionsrate Schutzmaßnahmen ggf. wieder zu intensivieren, um das Ausbruchsgeschehen beherrschbar zu halten. Ein Verständnis für diese Maßnahmen und eine ausreichende Mitwirkung aufseiten der Bevölkerung sind dabei erforderlich. Erkenntnisse darüber, wie die Bevölkerung die Empfehlungen und Verordnungen zur Infektionsvermeidung bisher wahrgenommen und warum sie diese teilweise nicht befolgt hat, können einen Beitrag dazu leisten, Maßnahmen zukünftig zielgruppensensibler zu kommunizieren und damit erfolgreicher zu implementieren. Vor diesem Hintergrund zielt die vorliegende Studie auf Basis der Beiträge in ausgewählten sozialen Medien darauf ab, die Einstellungen und Überzeugungen in Bezug auf die behördlich verordneten Infektionsschutzmaßnahmen zu untersuchen. Die Forschungsfrage der Studie lautet: Warum lehnen Teile der Bevölkerung behördliche Empfehlungen und Maßnahmen zum Schutz vor SARS-CoV‑2 und COVID-19 ab? Mit dem Begriff „behördliche Empfehlungen und Maßnahmen“ sind die im Zuge der COVID-19-Pandemie eingeführten Infektionsschutzmaßnahmen gemeint, die von Bund und Ländern implementiert wurden.

## Methodik

### Datenauswahl

3 soziale Netzwerke (Facebook, Twitter und der Kommentarbereich von Youtube) wurden hinsichtlich der Einstellungen und Überzeugungen über Social Distancing, das Tragen von Mund-Nasen-Bedeckungen und andere Schutzmaßnahmen untersucht. Dazu wurde in den sozialen Netzwerken jeweils u. a. nach den Stichworten „Corona“, „Corona-Virus“, „COVID-19“, „Corona-Maßnahmen“, „Corona-Schutzmaßnahmen“ und „SARS-CoV-2“ gesucht (Tab. 1).

**Table 1:** 

Soziales Netzwerk	Suchbegriffe und Hashtags	Filter
Youtube	„Corona“ „Coronavirus“ „Corona-Virus“ „COVID-19“ „SARS-CoV-2“ „Corona-Maßnahmen“ „Corona-Schutzmaßnahmen“	Videos sortieren nach: „Relevanz“ Kommentare sortieren nach: „Top-Kommentare“
Facebook	„Corona“ „Coronavirus“ „Corona-Virus“ „COVID-19“ „SARS-CoV-2“ „Corona-Maßnahmen“ „Corona-Schutzmaßnahmen“ „#corona“ „#coronavirus“ „#coronamassnahmen“ „#coronaschutzmaßnahmen“	Beiträge: „Öffentliche Beiträge“ Gruppen: „Öffentliche Gruppen“
Twitter	„Corona“ „Coronavirus“ „Corona-Virus“ „COVID-19“ „SARS-CoV-2“ „Corona-Maßnahmen“ „Corona-Schutzmaßnahmen“ „#corona“ „#coronavirus“ „#covid19“ „#sarscov2“ „#coronamaßnahmen“ „#coronaschutzmaßnahmen“	Sprache: „Deutsch“ Antworten: „Antworten und ursprüngliche Tweets aufnehmen“ Links: „Tweets mit Links aufnehmen“

Bei Youtube wurde die Option genutzt, die Ergebnisse der Suche nach Relevanz zu sortieren. Dabei geht es darum, inwiefern der Titel, die Beschreibung und der Inhalt der Videos passend zu den eingegebenen Suchbegriffen sind und welche Videos die meiste Interaktion hervorgerufen haben [26]. Die Kommentare der Youtube-Videos wurden jeweils nach „Top-Kommentaren“ sortiert, gemessen an der Zahl der „Likes“ (= „Gefällt mir“). Die untersuchten Youtube-Beiträge stammen zum größten Teil aus dem Kommentarbereich von Nachrichtensender- und Rundfunkkanälen wie „ZDF-heute Nachrichten“ oder „Bayerischer Rundfunk“, da solche Youtube-Kanäle am häufigsten nach der Stichwortsuche erscheinen und dort auch die meisten Diskussionen zu COVID-19 vorzufinden sind.

Bei der Analyse von Facebook-Daten wurden nur öffentliche Beiträge (Postings) einbezogen, die durch die zuvor genannten Suchbegriffe zu finden waren. In Twitter wurden Tweets und Antworten auf die ursprünglichen Tweets einbezogen, unabhängig davon, ob die Tweets einen hohen Interaktionsgehalt aufwiesen, oft „geliked“ oder retweetet wurden. Zudem wurde in Facebook und Twitter auch nach bestimmten Hashtags (Schlüsselwörter mit vorangestelltem Rautezeichen) gesucht, die in Tab. 1 dargestellt sind. Die Beiträge, die für die Forschungsfrage der Studie als besonders relevant erachtet wurden, wurden von einer Autorin des Beitrags (DW) aus den sozialen Netzwerken in ein Textdokument extrahiert. Eingeschlossen wurden Beiträge, die potenzielle Gründe für die Ablehnung behördlicher Schutzmaßnahmen thematisierten. Die Suche nach relevanten Beiträgen wurde solange durchgeführt, bis eine Informationssättigung erreicht war, d. h. sich durch die Extraktion weiterer Beiträge keine neuen Gründe für die Ablehnung behördlicher Maßnahmen ergaben.

Die Daten wurden im Zeitraum vom 09. bis zum 20.04.2020 abgerufen und beziehen sich auf deutschsprachige Beiträge, die Nutzer/innen im Zeitraum vom 02.03. bis 18.04.2020 vorgenommen haben. Die ersten Schutzmaßnahmen, die im März 2020 eingeführt wurden, motivierten die Wahl des genannten Erfassungszeitraums. Da die bundesweit geltenden Kontaktbeschränkungen bis zum 19.04.2020 galten und danach erste Lockerungen stattfanden [27], wurde der Zeitraum der Postings auf den 18.04.2020 begrenzt. Zudem wurde durch die zeitliche Begrenzung das Ziel verfolgt, die anfänglichen Einstellungen und Überzeugungen der Nutzer/innen sozialer Medien kurz nach der Einführung der behördlichen Maßnahmen zu erfassen.

### Datenanalyse

Insgesamt wurden 119 Beiträge, von denen 47 aus Youtube, 24 aus Twitter und 48 aus Facebook stammten, in die Analyse aufgenommen. Die extrahierten Beiträge wurden in Anlehnung an die inhaltlich strukturierende qualitative Inhaltsanalyse nach Kuckartz induktiv ausgewertet [28]. Die Beiträge wurden hierzu einzeln analysiert und relevante Passagen wurden im Textdokument markiert. Jeder Beitrag wurde entsprechend seiner Bedeutung von einer Autorin (DW) codiert. Textpassagen mit einer ähnlichen Codierung wurden zu Kategorien zusammengefasst. Zudem wurden inhaltlich zusammenhängende Unterkategorien den jeweiligen übergeordneten Hauptkategorien zugeordnet. Aus den Kategorien und Unterkategorien wurden anschließend Codes abgeleitet [29]. Die identifizierten Codes, Kategorien und Unterkategorien wurden schließlich durch alle Autor/innen gemeinsam überprüft und interpretiert. Etwaige Unstimmigkeiten wurden gemeinsam diskutiert bis schließlich ein Konsens hergestellt werden konnte.

## Ethische Aspekte

Die Untersuchung nutzt öffentlich verfügbare Informationen aus frei zugänglichen sozialen Netzwerken, die entsprechend dem Vorgehen aus früheren Untersuchungen als Open Data angesehen wurden [30, 31]. Um trotz der öffentlichen Verfügbarkeit der Daten die Anonymität der jeweiligen Nutzer/innen im Rahmen dieses Artikels zu gewährleisten, wurden die Nutzerbeiträge anonymisiert. Hierzu wurden die Namen der Beitragenden durch eine standardisierte Kennung („Nutzer/in“) ersetzt. Obwohl es in der qualitativen Forschung gängige Praxis ist, direkte Zitate unverändert als Belege zu verwenden, haben wir hier darauf verzichtet, da dies durch den Einsatz von Suchmaschinen ermöglichen würde, ggf. die Identität der ursprünglichen Autoren/innen zu ermitteln. Den gängigen Standards folgend wurden daher kleinere Änderungen an den Beiträgen vorgenommen, um die Anonymität zu gewährleisten. Diese Änderungen umfassen z. B. die Korrektur von Rechtschreibfehlern oder die Entfernung von Abkürzungen. In allen Fällen wurde sichergestellt, dass die inhaltliche Bedeutung der Beiträge weiterhin bestehen bleibt.

## Ergebnisse

Im Zuge der qualitativen Inhaltsanalyse der Beiträge konnten 6 Hauptkategorien und 4 Unterkategorien identifiziert werden (Tab. 2).

**Table 2:** 

Hauptkategorie	Unterkategorie	Anzahl der Postings
Fehlinformationen der sozialen Medien	Verharmlosung von COVID-19	44
	Zweifel an der Wirksamkeit von Schutzmaßnahmen	6
Misstrauen gegenüber den etablierten öffentlichen Medien	Keine	6
Wissensdefizite und Verunsicherung der Nutzer/innen sozialer Medien	Keine	7
Einschränkung der Grundrechte	Keine	9
Die Rolle der Regierungsvertreter und Behörden	Bevölkerungskontrolle durch Regierung	6
	Mangelndes Vertrauen in das Robert Koch-Institut	6
Wirtschaftliche Auswirkungen der Pandemie	Keine	26

## Fehlinformationen der sozialen Medien

### Verharmlosung von COVID-19

Die Auswertung zeigt, dass in den sozialen Medien zahlreiche Kommentare vorhanden sind, welche COVID-19 und die mit der Erkrankung verbundenen Risiken verharmlosen. Beispielsweise wird die Behauptung aufgestellt, dass getroffene Infektionsschutzmaßnahmen übertrieben seien und das Virus überschätzt würde. Maßnahmen seien aufgrund der geringen Zahl an Sterbefällen nicht gerechtfertigt und daher abzulehnen:

Vergleicht man diesen Virus mit dem SARS [auch Corona] aus den Jahren 2002/2003, so ist dieses bisher vergleichsweise harmlos …. Damals wurde nicht so eine Panik verbreitet. Heute hebt sich die Welt buchstäblich aus den Angeln. Diese Hysterie ist unbegründet und völlig überzogen. … Diese völlig überzogenen Abschottungsmaßnahmen schaden mehr, als sie nutzen … [Nutzer/in #59].

Die Zahlen der Infizierten rechtfertigen die Maßnahmen nicht. Es gibt weltweit täglich mehr Hungertote als die gesamte Pandemie Menschenleben gekostet hat … Ihr [gemeint sind die Befürworter der Schutzmaßnahmen] beklatscht gerade ein Verhalten des Staates, wogegen Menschen in der DDR auf die Barrikaden gegangen sind [Nutzer/in #89].

Viele Beiträge beschäftigen sich auch mit den jeweiligen Infektionswahrscheinlichkeiten und dem Risiko eines schweren Krankheitsverlaufes, wobei vor allem die höhere Anfälligkeit (Suszeptibilität) von Älteren und Personen mit Vorerkrankungen thematisiert wird.

Ebenso finden sich Bezüge zwischen der Letalität von COVID-19 und Influenza:

Aus Corona wird eine Massenhysterie erzeugt und fast alle Länder machen mit. Dabei hat dieses Virus in der Statistik eine null vor dem Komma und einige danach … Warum sind die jährlich in Deutschland fast 25.000 Toten durch Influenza nicht gesendet worden? Kein Wort war in den Medien zu hören. Aber in ein paar Monaten wird es auch der dümmste Deutsche begreifen, dass alles übertrieben war … [Nutzer/in #17].

So überbewertet, an der richtigen Grippe sterben mehr Menschen [Nutzer/in #48].

### Zweifel an der Wirksamkeit von Schutzmaßnahmen

Einige Personen stellen die Wirksamkeit implementierter Maßnahmen infrage:

Trotz der strengen Maßnahmen steht Bayern immer noch ganz oben in der Statistik [Nutzer/in #98].

Und wir werden weiter feststellen, dass die Fallzahlen weiter steigen werden. Und selbst wenn alle Türen zugemauert werden, bleibt das so [Nutzer/in #100].

All diese Verlängerungen [bezogen auf den Lockdown] bringen nichts. Corona bleibt auch, wenn alles zu ist [Nutzer/in #81].

## Misstrauen gegenüber den etablierten öffentlichen Medien

Einige Beiträge illustrieren das mangelnde Vertrauen gegenüber der Berichterstattung der etablierten öffentlichen Medien:

Das [die negativen Auswirkungen des Coronavirus bei Missachtung der Schutzmaßnahmen, Anm. d. Autoren/innen] sagen uns die Medien. Aber wer sagt mir, dass es richtig ist? Stimmt es denn, was sie verbreiten? Hinterfrag mal selbst! [Nutzer/in #4].

ARD und ZDF sind auch nur Marionetten in einem großen Spiel. Wieso lädt man mal nicht Experten, Wissenschaftlicher, Ärzte in die Talkshows ein, die den ganzen Maßnahmen und Statistiken skeptisch gegenüberstehen? Wo bleibt hier denn die Demokratie? [Nutzer/in #103].

Deutlich wird hierbei die kritische Haltung gegenüber diesen Medien und den dort transportierten Informationen zur Wirksamkeit der Schutzmaßnahmen.

## Wissensdefizite und Verunsicherung von Nutzer/innen sozialer Medien

Die Beiträge in den sozialen Medien offenbaren, dass das Gesundheitswissen im Zusammenhang mit SARS-CoV‑2 und COVID-19 teilweise gering und die Verunsicherung groß ist:

Weiß einer, wie es überhaupt dazu kam [gemeint ist der Ausbruch des Coronavirus]? Ich nicht [Nutzer/in #46].

Wäre es nicht mal wichtig von einem Lungenspezialisten zu hören, durch welche Maßnahmen man bei einer beginnenden Corona-Infektion die Wahrscheinlichkeit einer Lungenentzündung verringern kann? … [Nutzer/in #10].

Meine Mutter hat momentan ein Kratzen im Hals. Hat sie jetzt Corona oder ist das einfach eine Grippe? [Nutzer/in #116].

Bin mir nicht sicher, ob es wirklich eine Gefahr ist oder eine Panikmache. Kann es sehr schlecht einschätzen. Kann mir jemand sagen, wie hoch die Gefahr wirklich ist? [Nutzer/in #60].

## Einschränkung der Grundrechte

In einigen Beiträgen werden die Schutzvorkehrungen als Einschränkung der Grundrechte, wie z. B. der Versammlungsfreiheit oder der Berufsfreiheit, thematisiert:

Wir werden nach Strich und Faden getäuscht. Die Regierung beraubt uns Schritt für Schritt unserer Grundrechte auf Basis von total unsicheren Fakten und falschen Zahlen. … [Nutzer/in #12].

Erstaunlich, wie die Leute sich pauschal ihre Grundrechte nehmen lassen. Man muss bei solchen Einschränkungen schon differenzieren, z. B. nach Regionen, Berufen, Schutzmöglichkeiten usw. [Nutzer/in #72].

Eigentlich gelten die [Schutzmaßnahmen] gar nicht, weil Verordnungen, die gegen das Grundgesetz verstoßen, nichtig sind. Es sei denn, die Regierung hat den Notstand ausgerufen, dann gelten sie natürlich. Die Versammlungsfreiheit ist grundgesetzlich garantiert [Nutzer/in #99].

Deutlich werden hier die wahrgenommene Einschränkung der persönlichen Freiheit und das subjektive Gefühl, von der Regierung getäuscht zu werden. Dies knüpft an das zuvor genannte Misstrauen gegenüber den öffentlichen etablierten Medien an. Manche Beiträge gehen weiter und fordern gegen vermeintliche Eingriffe in die Grundrechte vorzugehen und die Maßnahmen nicht hinzunehmen:

Hoffe sehr, dass dagegen [gemeint ist das Kontaktverbot] ein paar Leute klagen. Ganz klar ein Eingriff in unsere Grundrechte [Nutzer/in #90].

## Die Rolle der Regierungsvertreter und Behörden

### Bevölkerungskontrolle durch Regierung

In einigen Beiträgen der Nutzer/innen wird behauptet, dass die Regierung absichtlich Panik hinsichtlich der Coronaerkrankung schüre, um Maßnahmen zur Bevölkerungskontrolle einzuführen:

Meine Freiheit lasse ich mir von euch [gemeint ist die Regierung] nicht weiter einschränken! Ihr spinnt doch. Diese Panikmache ist Absicht, um uns in Zwangsimpfungen zu bringen. Nicht mit mir! Keine Nadel, kein Chip kommt in meinen Körper! [Nutzer/in #68].

… Zwangsimpfung kommt bestimmt. Nur deswegen macht man aus dem Virus eine große Sache und die Zwangsimpfung wird gewünschte Nebenwirkungen haben. Bill Gates gehört zu dem Club der Personen, die anstreben, die Weltbevölkerung zu reduzieren. Man kann sich also vorstellen, was alles auf uns zukommt und wo das alles herkommt [Nutzer/in #58].

Das Virus wird erst weg sein, wenn die App installiert ist und wir gechipt und geimpft sind … [Nutzer/in #83].

### Mangelndes Vertrauen in das Robert Koch-Institut

Das Robert Koch-Institut und seine Empfehlungen werden von einigen Nutzer/innen kritisch beurteilt. Empfehlungen werden als unangemessen empfunden und Informationen mangels Durchführung eigener Studien des Robert Koch-Instituts zur Infektionshäufigkeit in Zweifel gezogen. Ferner wird unterstellt, dass die Quote der schwereren COVID-19-Erkrankungs- und Todesfälle verfälscht und eigentlich niedriger wäre. Dies wird auf die Untererfassung bei asymptomatischen Fällen zurückgeführt:

Über die Rolle des RKI wird man in jedem Falle spätestens hinterher reden müssen. So viel an Falschinformation und Untertreibung bis ins Lächerliche. Ein Leiter, der vor einer Woche öffentlich erklärt, dass er sich das Ausmaß vorab selber nicht vorstellen konnte, obwohl es seit Monaten Tag für Tag offensichtlicher wurde … Wow … ein Offenbarungseid … [Nutzer/in #71].

Das Robert-Koch-Institut führt keine wichtigen Studien durch, es wird auch keine Stichproben-Studie gemacht, um zu sehen, wie viele Menschen vielleicht schon infiziert sind, ohne es zu wissen. Das würde auch die Quote der schwereren Verläufe und auch Todesfälle komplett verändern (und zwar senken). Unglaublich alles [Nutzer/in #70].

## Wirtschaftliche Auswirkungen der Pandemie

Durch die COVID-19-Pandemie und die eingeführten Infektionsschutzmaßnahmen sehen einige Nutzer/innen die Wirtschaftsaktivität stark beeinträchtigt. Etliche Personen könnten aufgrund dieser Einschränkung ihren Berufen, vor allem im Dienstleistungssektor, nicht nachkommen und riskierten damit ungewollt einen wirtschaftlichen Nachteil.

Leider ist es für Millionen Arbeiter, Selbstständige, dem Einzelhandel und dem Mittelstand zu spät, sie werden keinen Fuß mehr fassen können. Die Schuldigen und Falschberater, einschließlich dieser Regierung, bleiben unbestraft [Nutzer/in #17].

Es gibt gerade genügend Leute, die vereinsamen, arbeitslos werden, die Miete nicht mehr zahlen können etc. [Nutzer/in #71].

Durch die Einschränkungsmaßnahmen werde die Wirtschaft geschwächt, einzelne Betriebe und Arbeitsplätze gefährdet. Man solle die Einschränkungen aufheben, damit die Bevölkerung wieder ihren alltäglichen Tätigkeiten und Berufen nachgehen könne. Dabei wird davon ausgegangen, dass die meisten Menschen sowieso vorsichtiger geworden wären und es reichen würde, sich in der Öffentlichkeit durch Mund-Nasen-Bedeckungen zu schützen. Andere schlagen vor, die Risikogruppen zu isolieren und die restlichen Personen von der Ausgangsbeschränkung zu befreien:

Beendet diesen irren Shutdown jetzt, nicht erst an Ostern. Ihr zerstört Existenzen wegen eines Virus, das durch keine Maßnahme der Welt mehr einzudämmen wäre. Isoliert die Alten und Kranken! [Nutzer/in #107].

Die Verfasser/innen der Beiträge befürchten, dass die Auswirkungen der Pandemie auf die Wirtschaft schwerer wiegen als die unmittelbaren Folgen für die Gesundheit der Bevölkerung. Das Virus könne man durch die Maßnahmen sowieso nicht bekämpfen und man solle es einfach hinnehmen und damit leben, wie mit allen anderen Risiken, die es im Leben gibt:

Mit dem Virus werden wir leben müssen, wie mit allen anderen Risiken im Leben auch! Die Wirtschaft zu schwächen und Arbeitsplätze zerstören und die Bürger zu bevormunden ist das Allerletzte! Schluss damit! [Nutzer/in #7].

## Diskussion

Die vorliegende Studie untersuchte anhand von Inhalten aus 3 sozialen Netzwerken, wie die Bevölkerung die Infektionsschutzmaßnahmen, die im Zuge der COVID-19-Pandemie in Deutschland implementiert wurden, wahrnimmt und was Gründe für die Nichteinhaltung bestehender Empfehlungen und Vorschriften sind. Die Untersuchung zeigt, dass die individuellen Einstellungen und Überzeugungen dabei durch 6 Dimensionen geprägt werden bzw. sich in diesen widerspiegeln.

In den sozialen Medien ist es einfach, Fehlinformationen zu verbreiten, wodurch Nutzer/innen auch leicht dazu veranlasst werden können, falsche Überzeugungen zu akzeptieren [32]. Da Online-Nutzer/innen zudem auch die Neigung haben, nach Informationen zu suchen, die ihre bereits bestehenden Überzeugungen bestätigen, und kontrastierende Informationen nicht zu beachten [33], können Personen, die ohnehin schon an den Maßnahmen zur Eindämmung des Virus gezweifelt haben, leichter von Fehlinformationen überzeugt werden. Dies lässt sich auch in den untersuchten Beiträgen der vorliegenden Studie erkennen.

Durch die Aufnahme von Informationen, welche COVID-19 und die mit der Erkrankung verbundenen Gefahren verharmlosen, und die Interpretation dieser kann die Ungewissheit der Bevölkerung bezüglich SARS-CoV‑2 und COVID-19 potenziell verstärkt werden. Zudem kann es zu einer Unterschätzung der COVID-19-Pandemie kommen, indem beispielsweise nur ältere Menschen und Personen mit Vorerkrankungen als Risikogruppe wahrgenommen werden und die Gefährdung anderer Personen vernachlässigt wird. Eine solche Verharmlosung kann eine typische Abwehrreaktion bei der Konfrontation mit Gesundheitsrisiken darstellen. Die Betonung des höheren Risikos eines schweren Verlaufs von COVID-19 bei vorerkrankten und älteren Menschen zeigt die Ablehnung einer persönlichen Betroffenheit von der Krankheit. Einige Nutzer/innen von sozialen Medien glauben demnach, dass sie mit einer geringeren Wahrscheinlichkeit negative Ereignisse erleben als andere Personengruppen („unrealistic optimism“; [15]). Zusätzlich werden durch den Vergleich mit Influenza oder weltweiten Hungersnöten Bedrohungen verharmlost und risikobehaftetes Verhalten gerechtfertigt. Die Verharmlosung von COVID-19 war in den sozialen Netzwerken am häufigsten vorzufinden und stellt somit eine Kernkategorie der vorliegenden Arbeit dar. Es ist jedoch zu berücksichtigen, dass die einbezogenen Postings aus der anfänglichen Phase der Pandemie stammen und mit zunehmender Dauer der COVID-19-Pandemie und mit steigender Anzahl an COVID-19-bedingten Todesfällen die Verharmlosung der Gefahren durch SARS-CoV‑2 abgenommen haben und damit die Relevanz dieser Subkategorie zurückgegangen sein kann.

Einen weiteren Abwehrmechanismus, welcher aus den Beiträgen der Nutzer/innen sozialer Medien zu entnehmen ist, stellt die Abwertung der Sender gesundheitsbezogener Botschaften dar [15]. Dem RKI und der Regierung werden negative Absichten, wie die Kontrolle der Bevölkerung durch Zwangsimpfungen sowie die Manipulation der Quote der COVID-19-Erkrankungs- und Todesfälle, unterstellt. Der Regierung und den Behörden wird somit ein mangelndes Vertrauen entgegengebracht, woraus eine Missachtung empfohlener Maßnahmen folgen kann.

Wissensdefizite im Zusammenhang mit SARS-CoV-2/COVID-19 können oben genannte Reaktionen verstärken [34]. Als deren Folge kann es zu einer falschen Wahrnehmung von Risiken kommen sowie ein Verhalten hervorrufen, bei dem Menschen selbst Schutzmaßnahmen auf Kosten anderer bewusst vernachlässigen, dabei aber selbst vom Infektionsschutz profitieren, der dadurch entsteht, dass andere die Schutzmaßnahmen befolgen. Eine Erhöhung des Wissens im Zusammenhang mit COVID-19 könnte dem entgegenwirken und einen Beitrag zur Förderung der sozialen Verantwortung leisten [35].

Die vorliegende Untersuchung zeigt, dass auch Misstrauen gegenüber der Berichterstattung in den etablierten öffentlichen Medien eine große Rolle in den Social-Media-Beiträgen spielt. Dies spiegeln die Ergebnisse von repräsentativen Umfragen wider, aus denen hervorgeht, dass ein großer Teil der Bevölkerung der Berichterstattung kritisch gegenübersteht [36].

Einige Personen nehmen die Infektionsschutzmaßnahmen als starke Beschränkung ihrer Grundrechte wahr. Vor allem in liberalen Gesellschaften wie Deutschland erscheinen solche Schutzmaßnahmen, anders als in Staaten wie China, die bereits vor der Pandemie sehr restriktiv waren, als große Einschränkung persönlicher und gesellschaftlicher Freiheit. Dies bestätigen auch Ergebnisse unterschiedlicher Bevölkerungsumfragen [37, 38]. Dies kann zur Folge haben, dass Maßnahmen als Ausdruck der Ablehnung restriktiver Eingriffe nicht befolgt werden. Studien zur Reaktanz [39] zeigen, dass Personen bei wahrgenommener Beschränkung ihrer individuellen Freiheit versuchen diese wiederherzustellen. Im aktuellen Fall kann dies Personen zu höherem Risikoverhalten im Zusammenhang mit COVID-19 provozieren [15] und zur Missachtung empfohlener Schutzmaßnahmen beitragen.

Infektionsschutzmaßnahmen wie die Einschränkung des Betriebs von Hotels, Gaststätten und Freizeiteinrichtungen leisten zwar einen maßgeblichen Beitrag dazu, die COVID-19-Pandemie beherrschbar zu halten, sie gehen jedoch auch mit negativen wirtschaftlichen Konsequenzen einher, deren Auswirkungen noch jahrelang wirtschaftlich spürbar sein werden [40]. Da viele Menschen bereits heute persönlich durch gewerbliche Einschränkungen betroffen sind, können Überzeugungen gefördert werden, dass die Kontaktsperre, die Ausgangsbeschränkungen und andere Schutzvorkehrungen abgeschafft werden sollten, da die wirtschaftlichen Konsequenzen die gesundheitlichen Nachteile überwiegen würden. Vor diesem Hintergrund ist es notwendig, bezüglich wirtschaftspolitischer Unterstützungsmaßnahmen transparent zu kommunizieren und überzeugend darzulegen, wie diese den pandemiebedingten ökonomischen Einschränkungen entgegenwirken.

Unseres Wissens ist dies die erste Studie in Deutschland, in der Gründe für die Ablehnung der Maßnahmen zur Beschränkung des öffentlichen Lebens im Zusammenhang mit SARS-CoV‑2 aus der Perspektive von Nutzern/innen sozialer Medien untersucht werden. Hierbei muss als Limitation jedoch berücksichtigt werden, dass als Datenquellen nur 3 häufig genutzte soziale Netzwerke ausgewählt wurden. Nutzer/innen sozialer Netzwerke in Deutschland sind vor allem 14- bis 49-Jährige [41] und häufiger weiblich [42]. Da das Nutzerprofil nicht repräsentativ für die gesamte Bevölkerung ist, ist unklar, inwiefern die Ergebnisse auch auf andere Bevölkerungsgruppen übertragbar sind. Ferner konnten bei der Auswertung der Beiträge auch die demografischen und sozioökonomischen Informationen der Nutzer/innen nicht einbezogen werden, da entsprechende Informationen fehlten. Zudem fand die Kodierung der einbezogenen Beiträge nur durch eine Autorin statt, wodurch Postings subjektiv interpretiert und mögliche andere Bedeutungsaspekte ausgeschlossen wurden. Die Erstellung von Codes und die Zuordnung der Beiträge zu Haupt- und Unterkategorien können demnach durch die individuelle Sichtweise der Autorin verzerrt sein. Gleichwohl wurden die identifizierten Codes, Kategorien und Unterkategorien durch alle Autor/innen gemeinsam überprüft und interpretiert.

Darüber hinaus war der Zeitraum der Postings auf den 18.04.2020 begrenzt. Da davon auszugehen ist, dass im Laufe der Pandemie die Ablehnung gegenüber behördlichen Maßnahmen zunimmt, sind möglicherweise relevante Ablehnungsgründe aufgrund der zeitlichen Beschränkung der Studie nicht berücksichtigt worden. Zukünftige Untersuchungen sollten ermitteln, wie sich die in Social-Media-Beiträgen dargelegten Meinungen nach dem 18.04.2020 weiterentwickelt haben.

## Schlussfolgerung

Die Ergebnisse der Studie gewähren einen Einblick, wie die Bevölkerung in Deutschland mit den Maßnahmen zur Beschränkung des öffentlichen Lebens im Zusammenhang mit SARS-CoV‑2 umgeht. Erkenntnisse hierzu können einen Beitrag leisten, bevölkerungsbezogene Maßnahmen zum Umgang mit Public-Health-Krisen zielgruppensensibler zu kommunizieren und damit erfolgreicher implementieren zu können. Die Ergebnisse zeigen, dass die Nutzer/innen sozialer Medien mit einer Vielzahl von Abwehrmechanismen auf die behördlichen Schutzmaßnahmen reagieren. Um diese zu reduzieren und eine breitere Akzeptanz im Hinblick auf die Maßnahmen herzustellen, sollten bei der Verkündung behördlicher Empfehlungen Bedrohungen der Gesundheit nicht stark betont werden. Zudem sollten sachliche Argumente und positive bestärkende Elemente in den Vordergrund gerückt werden, statt Angst und Furcht zu erzeugen, um so mögliche Reaktanzeffekte zu vermeiden [15]. Um Wissenslücken zu schließen, sollte eine umfassende Gesundheitsaufklärung veranlasst werden. Da das Vertrauen der Bevölkerung der Regierung und den Behörden gegenüber ein wichtiger Faktor für die Akzeptanz von Infektionsschutzmaßnahmen ist, sollte eine transparente und evidenzbasierte Kommunikation angestrebt werden. Mittlerweile liegen einige Erfahrungen zu effektiver Risikokommunikation bei der Eindämmung der COVID-19-Pandemie aus unterschiedlichen Ländern vor [43]. Um die Glaubwürdigkeit zu erhöhen, muss die Risikokommunikation dabei durch Einheitlichkeit, verständliche Sprache und Empathie gekennzeichnet sein und anstreben, die digitale Gesundheitskompetenz der Bevölkerung zu erhöhen. Hierdurch können auch Fehlinformationen in sozialen Medien besser erkannt und verarbeitet werden. Die vorliegende Studie unterstreicht damit auch die Relevanz qualitätsgesicherter Informationen in den sozialen Medien, um die Irreführung der Bevölkerung zu verhindern.
